# Protective effect of arachidonic acid and linoleic acid on 1-methyl-4-phenylpyridinium-induced toxicity in PC12 cells

**DOI:** 10.1186/1476-511X-13-197

**Published:** 2014-12-19

**Authors:** Kim San Tang

**Affiliations:** School of Pharmacy, Monash University Malaysia, Jalan Lagoon Selatan, 47500 Bandar Sunway, Selangor, Darul Ehsan Malaysia

**Keywords:** 1-methyl-4-phenylpyridinium, Arachidonic acid, Linoleic acid, Parkinson’s disease, PC12 cells, PPAR gamma

## Abstract

**Background:**

Parkinson’s disease is a neurodegenerative disorder that is being characterized by the progressive loss of dopaminergic neurons of the nigrostriatal pathway in the brain. The protective effect of omega-6 fatty acids is unclear. There are lots of contradictions in the literature with regard to the cytoprotective role of arachidonic acid. To date, there is no solid evidence that shows the protective role of omega-6 fatty acids in Parkinson’s disease. In the current study, the potential of two omega-6 fatty acids (i.e. arachidonic acid and linoleic acid) in alleviating 1-methyl-4-phenylpyridinium (MPP^+^)-induced cytotoxicity in PC12 cells was examined.

**Methods:**

Cultured PC12 cells were either treated with MPP^+^ alone or co-treated with one of the omega-6 fatty acids for 1 day. Cell viability was then assessed by using the 3-(4,5-dimethylthiazol-2-yl)-2,5-diphenyltetrazolium bromide (MTT) assay.

**Results:**

Cells treated with 500 μM MPP^+^ for a day reduced cell viability to ~70% as compared to control group. Linoleic acid (50 and 100 μM) significantly reduced MPP^+^-induced cell death back to ~85-90% of the control value. The protective effect could be mimicked by arachidonic acid, but not by ciglitazone.

**Conclusions:**

Both linoleic acid and arachidonic acid are able to inhibit MPP^+^-induced toxicity in PC12 cells. The protection is not mediated via peroxisome proliferator-activated receptor gamma (PPAR-γ). Overall, the results suggest the potential role of omega-6 fatty acids in the treatment of Parkinson’s disease.

## Background

Parkinson’s disease is a neurodegenerative disease that affects 1-2% of people above 65 years of age. In population over 85 years old, the prevalence of the disease increases to 3-5% [1]. The disease is being characterized by the progressive loss of dopaminergic neurons in the midbrain's substantial nigra [2]. The clinical motor symptoms that are associated with Parkinson’s disease are bradykinesia, involuntary tremor, postural instability, muscle weakness and rigidity [3].

PC12, a cell line derived from a pheochromocytoma of the rat adrenal medulla, is used widely as a model for catecholaminergic transmission. These cells are popular because they are able to synthesize and release catecholamines in a similar manner as dopaminergic neurons and adrenal medullary chromaffin cells [4]. Unlike chromaffin cells, PC12 cells only synthesize very small amount of adrenaline [5–7]. PC12 cells treated with 1-methyl-4-phenylpyridinium (MPP^+^), a neurotoxin that causes parkinsonism, were extensively used as a model of Parkinson’s disease *in vitro* [8–10]. MPP^+^ has been shown to induce apoptosis, dissipation of mitochondrial membrane permeability, and elevation of intracellular reactive oxygen species level in PC12 cells [11].

Omega-6 fatty acids are polyunsaturated fatty acids [12]. These fatty acids play a crucial role in growth development and brain function. There are several types of omega-6 fatty acids, and the key ones are linoleic acid and arachidonic acid. Linoleic acid can be obtained from diet, such as vegetable oil [13]. Linoleic acid cannot be synthesized by the body, and thus it is necessary to obtain linoleic acid from diet sources [14]. Therefore, linoleic acid is classified as one of the essential fatty acids. On the other hand, arachidonic acid is not considered as one of the essential fatty acids since the body can synthesize arachidonic acid from linoleic acid [15]. Meat, fish, and egg are the main dietary source of arachidonic acid [13, 16, 17].

To date, there is no direct evidence that shows the protective role of omega-6 fatty acids in *in vitro* Parkinson’s model. This is the first study to examine the protective role of linoleic acid and arachidonic acid and their potential interaction in a Parkinson’s disease model simulated by exposing PC12 cells to MPP^+^ neurotoxin.

## Methods

### Materials

PC12 cells were purchased from the American Type Culture Collection (ATCC, CRL-1721.1). Dulbecco's Modified Eagle Medium (DMEM), horse serum and fetal bovine serum were Gibco products of Life Technologies (Grand Island, NY, USA). Arachidonic acid, linoleic acid, methylthiazolyldiphenyl-tetrazolium bromide (MTT), ciglitazone and bisphenol A diglycidyl ether (BADGE) were obtained from Sigma-Aldrich (Malaysia). 96-well culture plates were purchased from Corning (Lowell, MA, USA).

### Cell culture

PC12 cells were grown in DMEM medium, containing 4.5 g/L glucose, supplemented with 10% horse serum and 5% fetal bovine serum. The cells were maintained at 37°C in an environment consisting of 95% air and 5% carbon dioxide. The medium was changed every other day. For the experiments, the cells were seeded at a density of 5 × 10^4^ cells per well in 96-well culture plates for an overnight before subjected to experimental treatment.

### Induction of cell death

Twenty-four hours after plating, MPP^+^ was used to induce death in PC12 cells. To examine the effect of omega-6 fatty acids, cultures were exposed to linoleic acid or arachidonic acid alone or with MPP^+^ for 1 day. These fatty acids were initially diluted in DMSO to a stock concentration of 200 mM and stored in -20°C before use.

### Cell viability assay

The protective effect of compounds on cell viability was assessed by using MTT conversion assay. The cells were incubated with MTT solution (final concentration, 0.5 mg/ml) in the dark for 4 h at 37°C. The dark-blue formazan crystals formed in intact cells were solubilized with isopropanol solution acidified with 0.1 N HCl. The optical density of each well was measured with a microplate reader at the test wavelength of 570 nm. Optical density is directly proportional to the number of living cells in culture. The data obtained were then expressed as percentage of viable cells relative to the untreated control group value.

### Statistical analysis

Each treatment was performed in duplicate or triplicate and each experiment was repeated at least three times. Statistical differences between experimental groups were determined by performing one-way analysis of variance (ANOVA) and the Newman-Keuls multiple comparison test. A level of P < 0.05 was considered statistically significant.

## Results

**Figure 1:**
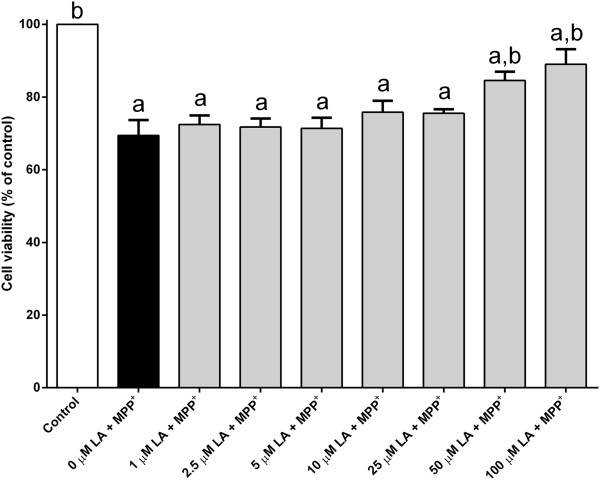
**Linoleic acid inhibits MPP** ^**+**^ **-induced cell death.** Cultured PC12 cells were subjected to 500 μM MPP^+^ in the absence or presence of different concentrations of linoleic acid (LA) (1–100 μM) for 24 h at 37°C. Number of viable cells was determined by MTT assay. The control was set to 100% survival. Data are means ± SEM (n = 3). Data were analyzed using one-way ANOVA and Neuman-Keuls’ test. Means with superscripts ^a^ and ^b^ are significantly different at P < 0.05 comparing control and 500 μM MPP^+^ only-treated groups, respectively.

In this study, MPP^+^ was employed as a tool to study the cell death. This compound causes loss of dopaminergic marker in the nigrostriatal neurons and a significant drop of dopamine level in the striatum of primates. Thus, MPP^+^-induce neuronal cell death is one of the common experimental models that is widely used to study the pathogenesis of Parkinson’s disease [8–10]. MPP^+^ is able to induce many pathological changes associated with cellular dysfunction such as neuronal cytoskeletal lesions, apoptosis, increases in mitochondrial permeability and intracellular calcium level. Treatment with MPP^+^ (500 μM) alone for 1 day yielded significant cell death to ~70% of control value (Figure 1).

**Figure 2:**
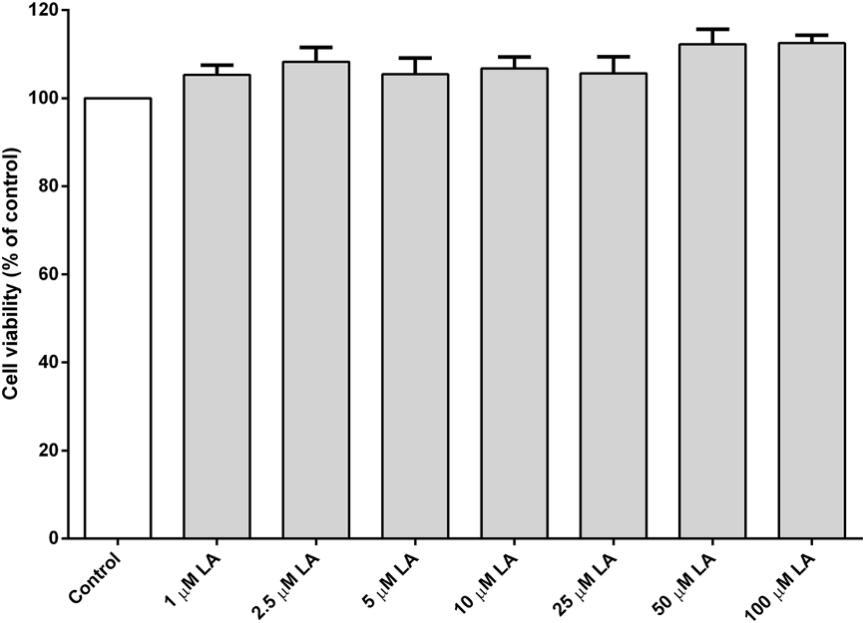
**The effect of linoleic acid on PC12 cells.** PC12 cells in culture were exposed to increasing doses of linoleic acid (LA) from 1–100 μM for 24 h at 37°C. Number of viable cells was determined by MTT assay. The control was set to 100% survival. Data are expressed as means ± SEM (n = 3). Data were analyzed using one-way ANOVA and Neuman-Keuls’ test. None of the data from treated-groups are statistically different when compared to the control.

Here, the potential protective role of two omega-6 fatty acids, namely arachidonic acid and linoleic acid was explored. PC12 cells were treated with MPP^+^ in the presence or absence of linoleic acid or arachidonic acid. As measured by MTT assay, MPP^+^-induced toxicity was attenuated in a dose-dependent manner when cultures were exposed to linoleic acid (1–100 μM) for 1 day (Figure 1). A significant amount of protection was observed in PC12 cells treated with linoleic acid (50–100 μM) when compared to MPP^+^-treated group (Figure 1). The cell viability was significantly increased to 85% and 90% of control value when treated with 50 μM and 100 μM of linoleic acid, respectively (Figure 1). Treatment with linoleic acid alone had no prominent effect on cell viability (Figure 2).

**Figure 3:**
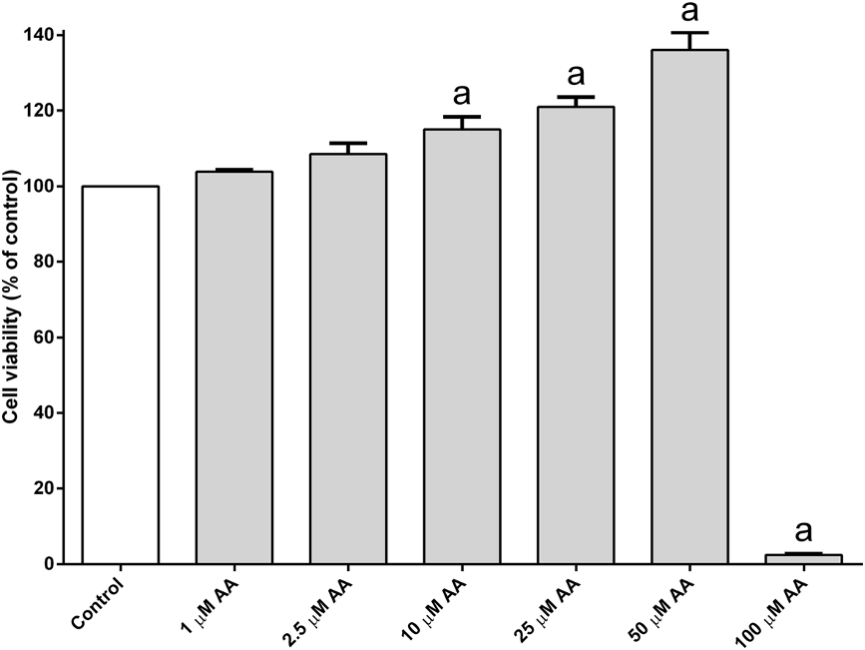
**The effect of arachidonic acid on PC12 cells.** Cultured PC12 cells were subjected to various concentrations arachidonic acid (AA) (1–100 μM) for 24 h at 37°C. Number of viable cells was determined by MTT assay. The control was set to 100% survival. Data shown are means ± SEM (n = 3). Data were analyzed using one-way ANOVA and Neuman-Keuls’ test. Means with superscript ^a^ are significantly different at P < 0.05 comparing control.

**Figure 4:**
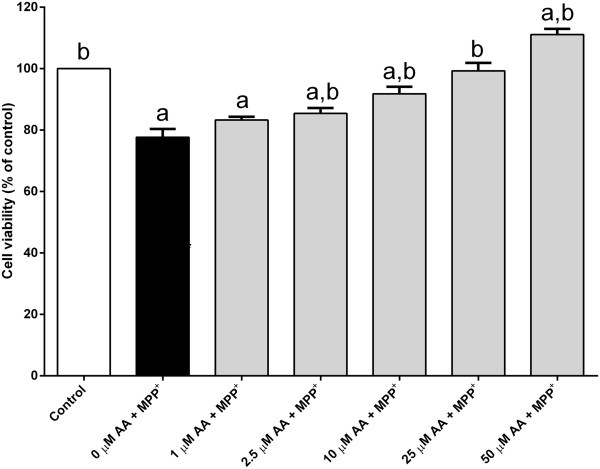
**Arachidonic acid attenuates MPP** ^**+**^ **-induced cell death.** PC12 cells were exposed to 500 μM MPP^+^ in the different doses of arachidonic acid (AA) (1–50 μM) for 24 h at 37°C. Number of viable cells was determined by MTT assay. The control was set to 100% survival. Data are expressed as means ± SEM (n = 3). Data were analyzed using one-way ANOVA and Neuman-Keuls’ test. Means with superscripts ^a^ and ^b^ are significantly different at P < 0.05 comparing control and 500 μM MPP^+^ only-treated groups, respectively.

**Figure 5:**
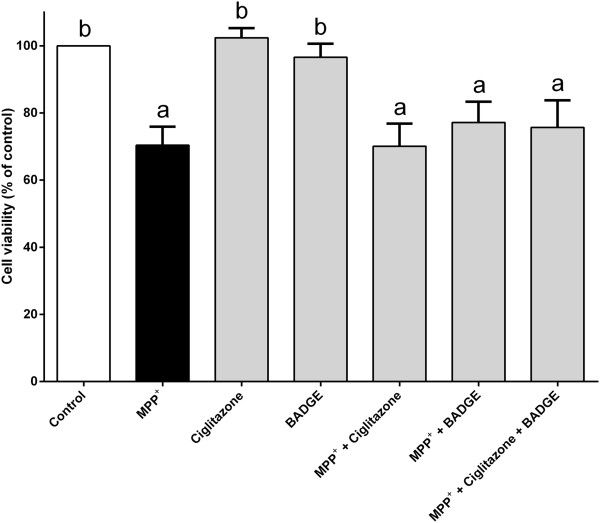
**Ciglitazone does not attenuate MPP** ^**+**^ **-induced cell death.** PC12 cells were treated with 500 μM MPP^+^, 1 μM ciglitazone, 10 μM BADGE, or combinations of these compounds for 24 h at 37°C. Number of viable cells was determined by MTT assay. The control was set to 100% survival. Data are means ± SEM (n = 3). Data were analyzed using one-way ANOVA and Neuman-Keuls’ test. Means with superscripts ^a^ and ^b^ are significantly different at P < 0.05 comparing control and 500 μM MPP^+^ only-treated groups, respectively.

**Figure 6:**
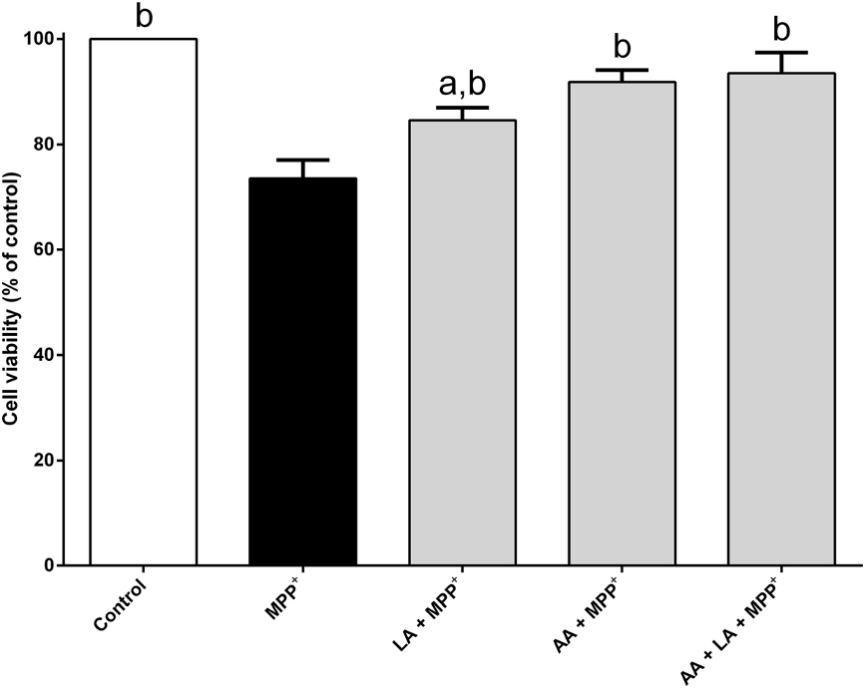
**Simultaneous treatment with arachidonic acid and linoleic acid.** PC12 cells were exposed to 500 μM MPP^+^ in the absence or presence of 10 μM arachidonic acid (AA), 50 μM linoleic acid (LA) or combination of both, for 24 h at 37°C. Number of viable cells was determined by MTT assay. The control was set to 100% survival. Data are means ± SEM (n = 3). Data were analyzed using one-way ANOVA and Neuman-Keuls’ test. Means with superscripts ^a^ and ^b^ are significantly different at P < 0.05 comparing control and 500 μM MPP^+^ only-treated groups, respectively.

The cell survival was enhanced when treated with arachidonic acid alone (Figure 3). Interestingly, a severe toxic effect was seen when the PC12 cells were treated with a high dose of arachidonic acid (Figure 3). The majority of PC12 cells were dead when treated with 100 μM of arachidonic acid. Despite of the high dose, treatment with arachidonic acid (25–50 μM) completely reverted MPP^+^-induced cell death back to or above the untreated control value (Figure 4). The protection is unlikely to be mediated via peroxisome proliferator-activated receptor gamma (PPAR-γ) since the effect could not be mimicked by agonist ciglitazone (1 μM) (Figure 5). Furthermore, the cell viability upon MPP^+^-treatment is slightly higher, although statistically insignificant, in the presence of a PPAR-γ antagonist, BADGE (10 μM). Although both arachidonic acid and linoleic acid show significant protection at 10 μM and 50 μM concentrations, respectively, simultaneous treatment of both fatty acids did not produce any additional protective effect against MPP^+^-induced cell death (Figure 6).

## Discussion

In the current work, MPP^+^-induced PC12 cell death was attenuated by two omega-6 fatty acids, namely linoleic acid and arachidonic acid. The latter is a type of polyunsaturated fatty acids, which is found mainly in brain tissues. It has many physiological roles, for example, blood clotting, brain development and can act as a signaling molecule in the brain [18–20]. The role of arachidonic acid on cytoprotection is rather controversial from the literature. The enzymatic oxidation of arachidonic acid produces pathological inflammatory mediators such as prostacyclins, thromboxane A2 and leukotrienes [21]. Arachidonic acid has been shown to cause apoptosis and cell death in many different cell types [22–25]. Many studies reported that arachidonic acid is cytotoxic even at concentartions of 50–100 μM in most cell lines [26]. Concentrations of arachidonic acid above 100 μM are able to induce necrosis in a variety of cells. Moreover, intravenous injection of high dose of arachidonic acid has been shown to cause sudden death in rabbit [27]. However, it is uncertain if arachidonic acid has any direct effect on cell viability on *in vivo* study. In this study, high dose of arachidonic acid (100 μM) has been shown to cause severe toxicity to cultured PC12 cells (Figure 3).

Despite this, concentrations of arachidonic acid up to 50 μM are protective against MPP^+^ insult in these cells (Figure 4). In addition, treatment with arachidonic acid alone improves the percentage of viable PC12 cells above the control value. The results of this study are consistent with several other reports demonstrating that arachidonic acid is indeed protective. For example, arachidonic acid can protect rat hippocampal slices against oxidative stress induced by hydrogen peroxide and glutamate [28]. Furthermore, arachidonic acid can also protect rat cardiac myocytes and gastric mucosa against ischemic and ethanol insults, respectively [29, 30].

Astrocytes are the major source of arachidonic acid in the brain [31–33]. Astrocytes can synthesize and release arachidonic acid. Astrocytes and neurons are located very close to one another. Thus, astrocytes are able to supply neurons with arachidonic acid. Astrocytes play significant roles in maintaining neuronal function and survival in the brain. The protective roles of astrocytes depend mainly on the substances that they release into and take up from the extracellular space, a microenvironment that is shared between astrocytes and neurons. In Parkinson’s disease, the role of astrocytes might be impaired. For example, MPP^+^ has been demonstrated to induce toxicity in astrocyte culture. MPP^+^ causes failure of energy metabolism in astrocytes and therefore, impairs the glutamate uptake capacity of these cells [34]. High level of extracellular glutamate can cause excitotoxicity to the surrounding neurons [35–37]. In addition to that, a lack of neuronal supply of arachidonic acid could also be one of the factors that causes neuronal death in Parkinson’s disease. Hence, further studies could be carried out to elucidate the effect of endogenous arachidonic acid by either using pre-conditioned media or astrocyte co-cultures.

The critical micelle concentration (CMC) values reported for arachidonic acid and linoleic acid are quite similar (~10-60 μM) [26, 38], hence concentrations above this level could form micelles that reduce the effectiveness of the compound when introduced into the medium. Despite of the potential limitation, both arachidonic acid and linoleic acid were found to be able to protect against MPP^+^-induced cell death in a dose-dependent manner (Figures 1 and 4). In addition, a significant amount of protection started to be seen when the cells treated with 2.5 μM of arachidonic acid, a concentration below its CMC value (Figure 4). Although some concentrations used here were above the CMC, biophysical analyses of fatty acids in water showed that micelles were rarely formed below pH 9.0 [39]. In fact, the pH of culture medium used in this study was maintained as close to the physiological pH as possible using CO_2_. Furthermore, the bicarbonate presents in the culture medium is a weak buffer and is able to prevent drastic pH changes. Bicarbonate is not only a non-toxic substance, but has nutritional value.

In addition to cytoprotection, arachidonic acid also exhibits growth-promoting effects on PC12 cells. The optical density obtained from MTT assay is directly proportional to the cell viability provided that there is no any other inference substance in this assay. Micelle formation is likely to be increased when higher concentrations of fatty acids are used. Therefore, one may argue that the formation of micelles may interfere with the optical density measurement of MTT assay and thus produces a false dose-dependent increase in cell viability as seen in arachidonic acid treatment (Figure 3). However, this is unlikely to be the case because the trend was not obvious when the cells were treated with increasing concentrations of linolenic acid, a compound with similar CMC value as arachidonic acid (Figure 2).

It has also been reported that arachidonic acid concentration in the cerebrospinal fluid is ~1 μM [40]. However, due to the close anatomical proximity between neurons and astocytes, it is not surprising for the neurons to be instantly exposed to high local concentration of arachidonic acid physiologically beyond the level that was measured from cerebrospinal fluid. Thus, the concentrations used in this study could be physiologically relevant and it is not unrealistic to see such a protection at these concentrations.

PPAR-γ is a nuclear receptor which acts as a transcriptional regulator of multiple genes and subsequently promoting cell differentiation, proliferation and survival in many cell types. It has been reported that PPAR-γ is present in PC12 cells [41, 42]. Previous studies have shown that arachidonic acid and its metabolites such as prostaglandins can activate PPAR-γ [43]. The results from this study show that ciglitazone, a PPAR-γ agonist, could not protect the PC12 cells from MPP^+^-induced death (Figure 5). Thus, the involvement of PPAR-γ signalling pathway in cytoprotection can be excluded from this study.

Linoleic acid has been shown to be able to protect necrotic and apoptotic cell death induced by palmitic acid [44]. In this study, linoleic acid has also been shown to reduce cell death in PC12 cells induced by MPP^+^. Linoleic acid is the precursor for the synthesis of arachidonic acid [45]. Most mammals, including human are able to convert linoleic acid to arachidonic acid [15]. The metabolic conversion occurs mainly in liver and brain, and is mediated by delta-5 and delta-6 desaturase [46]. In fact, these enzymes have been shown to be present in various mammalian cells and tissues [47, 48]. Interestingly, the metabolic conversion of linoleic acid to arachidonic acid has also been demonstrated in many cultured mammalian cells [49, 50]. Nonetheless, the possibility that the protective effect of linoleic acid is achieved via the *in vitro* metabolic conversion pathway cannot be excluded. These two fatty acids are likely to share and mediate the same downstream protective mechanisms since no additional protection against MPP^+^-induced cell death was seen when PC12 cells were simultaneously treated with linoleic acid and arachidonic acid (Figure 6).

## Conclusions

In conclusion, both linoleic acid and arachidonic acid can effectively protect PC12 cells against toxicity caused by MPP^+^. However, further studies are required to elucidate the protective pathway of these fatty acids. For instance, the mechanisms that lead to cellular toxicity such as antioxidant, anti-inflammatory and anti-apoptotic properties shall be explored further. The findings from this study suggest that omega-6 fatty acids could have a potential therapeutic or preventive role in Parkinson’s disease.

## Abbreviations

MPP^+^: 1-methyl-4-phenylpyridinium
MTT: 3-(4,5-dimethylthiazol-2-yl)-2,5-diphenyltetrazolium bromide
ANOVA: Analysis of variance
BADGE: Bisphenol A diglycidyl ether
CMC: Critical micelle concentration
PPAR-γ: Peroxisome proliferator-activated receptor gamma.
